# Applications of Ni-Based Catalysts in Photothermal CO_2_ Hydrogenation Reaction

**DOI:** 10.3390/molecules29163882

**Published:** 2024-08-16

**Authors:** Zhimin Yuan, Xianhui Sun, Haiquan Wang, Xingling Zhao, Zaiyong Jiang

**Affiliations:** 1 School of Chemistry & Chemical Engineering and Environmental Engineering, Weifang University, Weifang 261061, China; 2 Food and Drug Department, Weifang Vocational College, Weifang 261061, China

**Keywords:** photothermal catalysis, Ni-based catalysts, CO_2_ hydrogenation, photothermal catalysts

## Abstract

Heterogeneous CO_2_ hydrogenation catalytic reactions, as the strategies for CO_2_ emission reduction and green carbon resource recycling, play important roles in alleviating global warming and energy shortages. Among these strategies, photothermal CO_2_ hydrogenation technology has become one of the hot catalytic technologies by virtue of the synergistic advantages of thermal catalysis and photocatalysis. And it has attracted more and more researchers’ attentions. Various kinds of effective photothermal catalysts have been gradually discovered, and nickel-based catalysts have been widely studied for their advantages of low cost, high catalytic activity, abundant reserves and thermal stability. In this review, the applications of nickel-based catalysts in photothermal CO_2_ hydrogenation are summarized. Finally, through a good understanding of the above applications, future modification strategies and design directions of nickel-based catalysts for improving their photothermal CO_2_ hydrogenation activities are proposed.

## 1. Introduction

With the continuous progress of social productivity and science and technology, economic pillar industries such as chemical production, power systems, automobile manufacturing, etc., have experienced rapid development. This has been accompanied by massive consumption of fossil fuels and excessive emissions of the greenhouse gas CO_2_ [1,2,3,4,5,6,7,8]. The scientific problem of how to reduce CO_2_ emissions to the maximum extent has aroused great attention from all countries and governments, and they have put forward many coping strategies. Many countries have signed provisions such as the Kyoto Treaty and the Paris Agreement to control CO_2_ emissions and slow the pace of global warming [9].

In view of the serious impact of the greenhouse gas CO_2_ on the global climate and in order to alleviate human dependence on limited fossil energy, scientists have proposed to catalyze CO_2_ into high-value-added valuable organics to replace limited fossil resources and reduce the concentration of CO_2_ in the atmosphere [10,11,12,13,14,15]. Due to the high symmetry and chemical inertness of CO_2_ molecules, it is necessary to input a large amount of energy to dissociate the C=O double bond in the converting process of CO_2_; so, the introduction of catalysis is imperative. In recent years, many CO_2_ utilization technologies have been explored and discovered, mainly including photocatalysis, thermal catalysis, electrocatalysis, biological conversion, photothermal catalysis and other methods [16,17,18,19,20,21,22]. Photocatalytic CO_2_ technology converts CO_2_ into methane, carbon monoxide, methanol and other substances under the action of light and semiconductor catalysts. Thermal catalytic conversion of CO_2_ converts CO_2_ into useful chemicals or fuels by using catalysts under high-temperature conditions. Electrocatalysis refers to the conversion and reduction of CO_2_ into renewable energy under the action of electric energy and electrodes. Biological conversion is the conversion of CO_2_ into organics and fuels through metabolic methods and other means such as biological methods. Photothermal catalysis of CO_2_ is a new direction of catalytic conversion driven by solar energy and heat utilized in recent years. Photothermal catalysis includes four types of actions: thermal-assisted photocatalysis (thermal energy improves the separation of photo-generated charge carriers), photo-assisted thermocatalysis (solar energy enhances the local temperature of the catalyst surface), photo-driven thermocatalysis (the catalyst has high light absorption capacity, thereby achieving photo-to-thermal conversion under light irradiation) and photothermal co-catalysis (the catalyst could exhibit contributions to both the thermochemical and photochemical reactions) [23]. Different from thermal catalysis and photocatalysis, it collaboratively drives the catalytic reaction of CO_2_ by combining thermocatalysis and photocatalysis, which can usually enhance catalytic activity and selectivity compared to thermally driven reactions [24,25]. Among the above-mentioned CO_2_ conversion technologies, photothermal catalytic CO_2_ hydrogenation technology is regarded by the majority of scientific researchers as a technology with great application potential, and has received more and more attention [26,27,28,29,30].

Therefore, a large number of CO_2_ hydrogenation photothermal catalysts have been explored one after another, showing a very good industrial application prospect in terms of activity. Among them, supported catalysts (metal/oxide) are the hot-topic materials in this field, which are mainly composed of active metals (such as Rh [31], Ru [32,33], Pd [34], Pt [35,36], Cu [37,38,39], Ni [40], Co [41,42], Fe [43,44], etc. [45]) and metal oxide supports (for instance TiO_2_ [46,47,48], SiO_2_ [49], In_2_O_3_ [50],CeO_2_ [51,52], Al_2_O_3_ [53], etc. [54,55,56]). Taking into account the high price of precious metals, insufficient reserves and other problems, their large-scale promotion and industrialization is severely limited. From the perspective of large-scale industrial applications, non-precious metals with abundant reserves, cheap costs and good activity (for example, Cu, Ni, Co, etc.) have received ever-increasing attention [57,58,59,60,61,62,63,64,65,66]. And their explorations in the field of photothermal CO_2_ hydrogenation have gradually deepened in recent years [67,68,69,70,71,72,73,74,75].

Ni (nickel), despite being one of the hot-topic materials of non-precious metals, to date, there are few review papers reporting on applications of Ni-based catalysts in photothermal CO_2_ hydrogenation. To facilitate a deeper and more convenient comprehension for a broader audience of researchers in this field, it is imperative to conduct a comprehensive application review based on recent advances. This review will sum up the photothermal CO_2_ hydrogenation applications of Ni-based catalysts in three aspects: photothermal CO_2_ methanation, photothermal reverse water–gas reaction (RWGS), and photothermal CO_2_ hydrogenation to produce methanol. Based on the above applications, this review presents the research progress, some current problems and challenges, and some strategies to further enhance the activities Ni-based catalysts, and proposes future research directions.

## 2. Possible Photothermal CO_2_ Hydrogenation Reaction Path

The photothermal CO_2_ hydrogenation reaction of Ni-based catalysts is a complex multi-step reaction, so its reaction path is complex and uncertain, resulting in the formation of many types of products, such as CO, CH_4_ and CH_3_OH. The following is a possible and brief statement about the reaction path according to the different products [76,77,78,79].

Photothermal reverse water–gas shift reaction (CO_2_ + H_2_ ⇌ CO + H_2_O). The level of hydrogen consumption in the photothermal reverse water–gas shift reaction (RWGS) is the least out of all of the CO_2_ hydrogenation reactions. The CO product is the basic raw material for many important industrial products, so it is considered a highly popular reaction. The RWGS reaction includes two main reaction pathways: (1) the CO path. Carbon dioxide is first adsorbed on the surface of the catalyst to form *CO_2_ active species. Then, through the direct break of the C-O chemical bond, *CO_2_ can be directly converted into *CO and *O. Finally, *CO adsorbed on the catalyst surface produces CO via desorption. (2) The carboxyl (*HOCO) intermediate path. In this process, the CO_2_ adsorbed on the catalyst surface (*CO_2_) is hydrogenated to form the intermediate species carboxyl group (*HOCO), and then, the *HOCO intermediates are cleaved to form *CO and *OH species at the corresponding active sites. Finally, the *CO intermediates are desorbed from the catalyst surface to form gaseous CO. Regardless of the path, the adsorption capacity of *CO on the catalyst surface has an important effect on the selectivity of CO. A suitable adsorption capacity is conducive to the formation of CO. A strong adsorption capacity is not favorable for the desorption of *CO, which will cause *CO to continue under hydrogenation, thereby changing the selectivity of CO.

Photothermal CO_2_ methanation (CO_2_ + 4H_2_ ⇌ CH_4_ + 2H_2_O). CO_2_ methanation is one of the important reactions to produce methane, which has important practical significance in industrial production. It has been reported that there are two main reaction paths for photothermal CO_2_ methanation. The direct cleavage path of the C-O bond. CO_2_ is adsorbed on the surface of the catalyst to form *CO_2_ active species, which are directly converted into *CO and *O. The formed *CO continues to dissociate to *C and *O, and subsequently, *C can undergo hydrogenation to produce *CH, *CH_2_, *CH_3_ and *CH_4_. *CH_4_ is desorbed from the surface of the catalyst to form CH_4_. Alternatively, there is the formate pathway. *CO_2_ is hydrogenated to produce H_x_CO active species, and then, the C-O chemical bonds in H_x_CO are broken to form *CH_x_. *CH_x_ can continue to undergo a series of hydrogenation processes to form *CH_4_, which is subsequently desorbed from the catalyst surface to form CH_4_.

Photothermal methanol production (CO_2_ + 3H_2_ ⇌ CH_3_OH + H_2_O). As we all know, methanol is an important chemical raw material and hydrogen storage material, which makes the research on photothermal catalytic CO_2_ hydrogenation for methanol production the object of much attention. According to research reports, there are two main reaction paths for photothermal CO_2_ hydrogenation for methanol production at present. (1) The *CO intermediate path. *CO_2_ first undergoes the RWGS reaction to produce *CO, which can be continuously hydrogenated to produce CH_3_OH. (2) The formate pathway (*HCOO). *CO_2_ is hydrogenated to produce *H_2_COOH, which can be converted to CH_3_OH by breaking the C-O chemical bond and undergoing hydrogenation. It seems that the description is very simple, but in fact, the whole reaction process is complex and may also involve the conversion of many other intermediates.

## 3. Photothermal CO_2_ Hydrogenation Applications of Ni-Based Catalysts

### 3.1. Photothermal Reverse Water–Gas Reaction

The photothermal reverse water–gas reaction is a promising candidate for efficient utilization of CO_2_ and hydrogen energy. However, the conversion rate of carbon dioxide is limited by the thermodynamic equilibrium. It is a hot topic to explore photothermal catalysts with high activity, high CO selectivity and high CO_2_ conversion. In the early stage, Pt, Pd and some precious metals were found to have good catalytic activities in the RWGS reaction [36]. In recent years, considering the price and reserves of precious metals, the exploration of non-precious metal catalysts has become more and more extensive [80]. Among them, Ni-based catalysts have attracted great interest because of their abundant reserves, low price and good catalytic activity [81].

**Figure 1:**
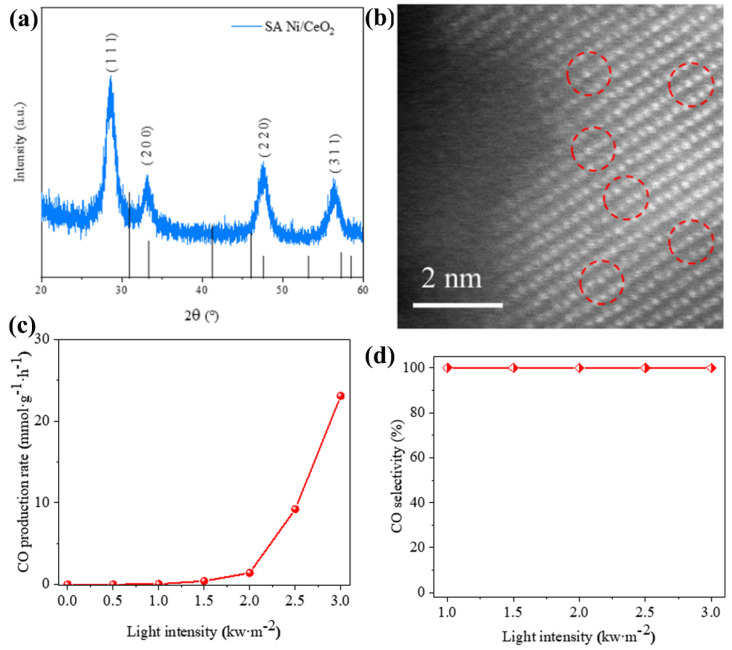
(**a**) The XRD image and (**b**) TEM image of atomic Ni/CeO_2_. (**c**) The CO production rate and (**d**) CO selectivity of atomic Ni/CeO_2_ under varying sunlight irradiation. Reprinted with permission from Ref. [82]. Copyright 2023, Elsevier B.V.

However, after more than ten years of exploration, it has been found that the CO selectivity of Ni-based catalysts is not high when performing RWGS, and there is always a large number of CH_4_ by-products which interfere. A lot of effort has been put into exploring the high selectivity of CO. For example, Song et al. discovered that single atoms of Ni can cause Pauli incompatibility through theoretical calculations, thereby preventing the formation of CO by-products in the process of RWGS [82]. Based on their calculations, in this study, nickel was loaded onto the surface of CeO_2_ nanosheets to explore the catalytic activity and selectivity of photothermal CO_2_ hydrogenation. By XRD characterization (Figure 1a), no diffraction peaks of Ni were found, indicating that Ni particles are very small. It was determined by TEM (Figure 1b) that Ni particles belong to the atomic level. Atomically, Ni/CeO_2_ underwent the photothermal RWGS reaction, and the results are shown in Figure 1c,d. It is evident that atomically, Ni/CeO_2_ exhibits 100% CO selectivity and has a good catalytic rate (23.1 mmol·g^−1^·h^−1^). By analyzing the XPS spectra of the samples before and after the RWGS reaction, it is found that the valence state of Ni is stable at the oxidation state +2 valence. It can be seen that the Ni active species on the catalyst surface have an important effect on the CO selectivity of the RWGS reaction.

**Figure 2:**
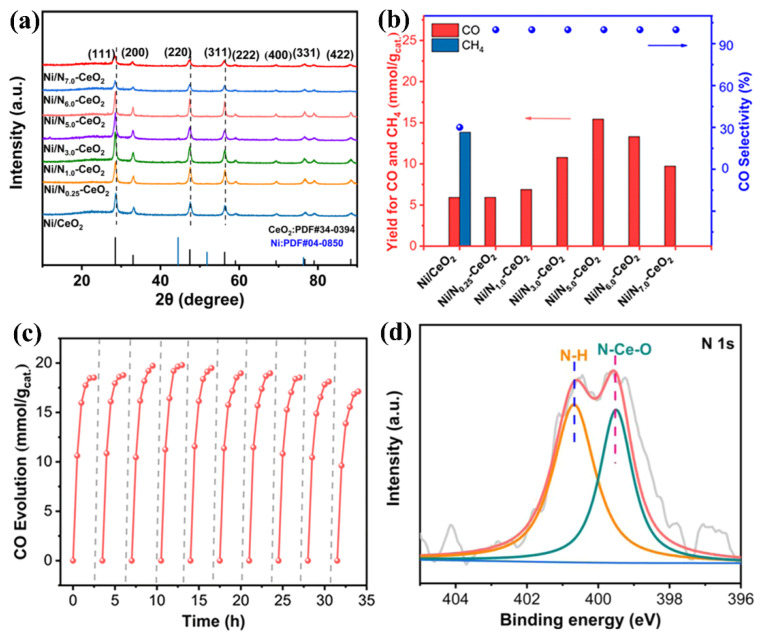
(**a**) XRD images of samples, (**b**) photothermal CO_2_ hydrogenation activities of samples, (**c**) cyclic reaction of Ni/N_5.0_-CeO_2_, and (**d**) XPS spectrum of N 1s in Ni/N_5.0_-CeO_2_ after reaction. Reprinted with permission from Ref. [83]. Copyright 2021, American Chemical Society.

In addition, the support of Ni-based catalysts also has an important effect on the RWGS reaction. For example, researchers have explored significant differences between CeO_2_ and N-doped CeO_2_ [83]. Based on the XRD image (Figure 2a), it can be found that the diffraction peak of Ni/CeO_2_ is shifted to a lower angle after nitrogen doping, which confirms the successful doping of N. The photothermal catalytic CO_2_ hydrogenation activity diagrams (Figure 2b) show that the CO selectivity of Ni/CeO_2_ is only 30%, while the CO selectivity of Ni/N_x_-CeO_2_ is almost 100%. And the CO yields of the Ni/N_x_-CeO_2_ samples are improved compared to those of Ni/CeO_2_. In addition, the cyclic reaction exhibited that the catalytic activity has no decrease (Figure 2c), suggesting that the Ni/N_x_-CeO_2_ composite possesses excellent stability. Through XPS (Figure 2d) and in situ FT-IR characterization, the researchers found that N-H chemical bonds appear on the surface of Ni/N_x_-CeO_2_ after the photothermal RWGS reaction, which reduces the number of active *H species. It was found by theoretical calculation that the ability of Ni/N_x_-CeO_2_ to break down hydrogen is weaker than that of Ni/CeO_2_. These results indicate that changes in selectivity are significantly associated with the formation of N-H chemical bonds. The Gibbs free energy calculation results show that *CO is more easily desorbed from the surface of Ni/N_x_-CeO_2_ than Ni/CeO_2_, thereby being more conducive to CO generation. Moreover, the alloying strategy is also an effective method to increase CO selectivity in the photothermal CO_2_ hydrogenation reaction of Ni-based catalysts. The researchers found that a Ni-Mo alloy can regulate the selectivity of CO in photothermal CO_2_ hydrogenation, and even Ni1Mo1 can obtain 98% CO selectivity [77]. The studies show that Mo can regulate the electronic structure of Ni, weaken the ability of Ni to decompose hydrogen and increase the desorption of *CO from the surface of the catalyst, which is the main cause of the formation of CO.

### 3.2. Photothermal CO_2_ Methanation

In 1897, Paul Sabatier experimentally confirmed the CO_2_ methanation reaction. After more than 120 years of exploration and research, CO_2_ methanation (that is, the Sabatier reaction) technology has been better expanded and developed. CH_4_ (methane) is a key component of natural gas, which is a high-quality energy source and has great potential to replace coal as a clean fuel. In addition, it can also be used as a chemical raw material for the production of acetylene, carbon black, methane chloride and other chemical products. Therefore, it has gradually become a strategic resource and aroused widespread interest globally among researchers. Currently, thermal catalytic CO_2_ methanation has been used commercially on a large scale [84]. However, due to the high stability of CO_2_ molecules, high temperature and high pressure are needed to achieve efficient conversion, which leads to high energy consumption, environmental problems and harsh reaction conditions. It is of great significance to use renewable and clean energy to promote CO_2_ methanation under milder and more environmentally friendly conditions. Solar energy is a non-polluting, sustainable renewable energy source, and has been regarded as a viable auxiliary thermal catalytic CO_2_ methane energy source. Therefore, photothermal catalytic CO_2_ methanation technology has become one of the hot technologies because of its advantages such as mild reaction conditions, low energy consumption, green environmental protection capacity and good performance [84]. A large number of CO_2_ methanation photothermal catalysts have been explored, showing a very good prospect of industrial application in terms of activity. In particular, Ni-based catalysts have been considered promising materials and have been extensively explored [85,86].

**Figure 3:**
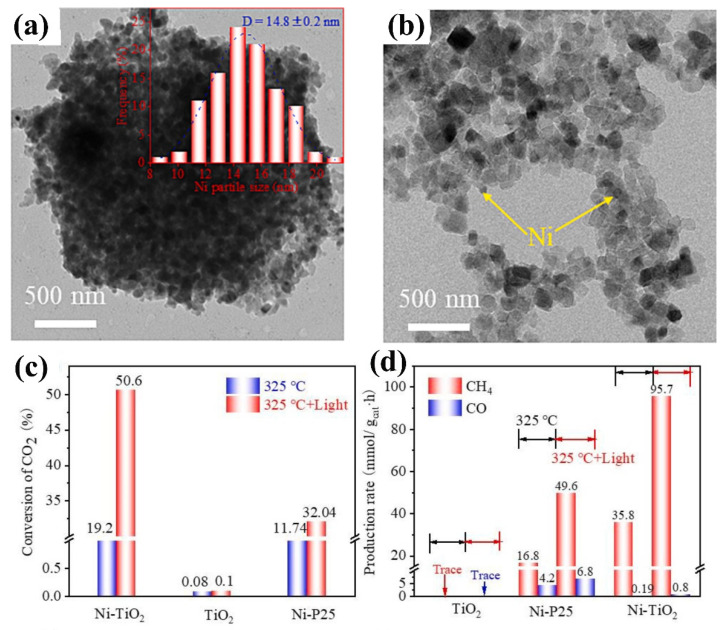
(**a**,**b**) The TEM image of Ni/TiO_2_. (**c**) The CO_2_ conversion and (**d**) production rate of the samples [87]. Copyright 2023, Elsevier B.V.

For instance, Li et al. obtained TiO_2_ with a large specific surface area by converting MIL-125 (Ti-MOFs) and then loaded Ni particles with a smaller size and better dispersion on its surface, compared to using P25 (Figure 3a,b) [87]. Through the photothermal CO_2_ methanation reaction (Figure 3c,d), it can be seen that the catalytic activity of pure TiO_2_ is very low, and the introduction of nickel can greatly improve its catalytic activity. In addition, Ni/TiO_2_ exhibits a higher CO_2_ conversion rate than Ni/P25, and its methane selectivity is close to 99%. A series of characterization tests confirmed that a large specific surface area is conducive to the adsorption and activation of carbon dioxide, and highly dispersed nickel particles can provide more surface-active sites for CO_2_ methanation. Ni/TiO_2_ has stronger interfacial interactions than Ni/P25, thereby increasing the density of Ni electronic states on the surface of Ni/TiO_2_, which is conducive to improving the ability of Ni to decompose H_2_ and CO_2_. It can be seen from this work that the size, dispersion and surface electron state density of nickel particles play important roles in the photothermal CO_2_ methanation reaction.

**Figure 4:**
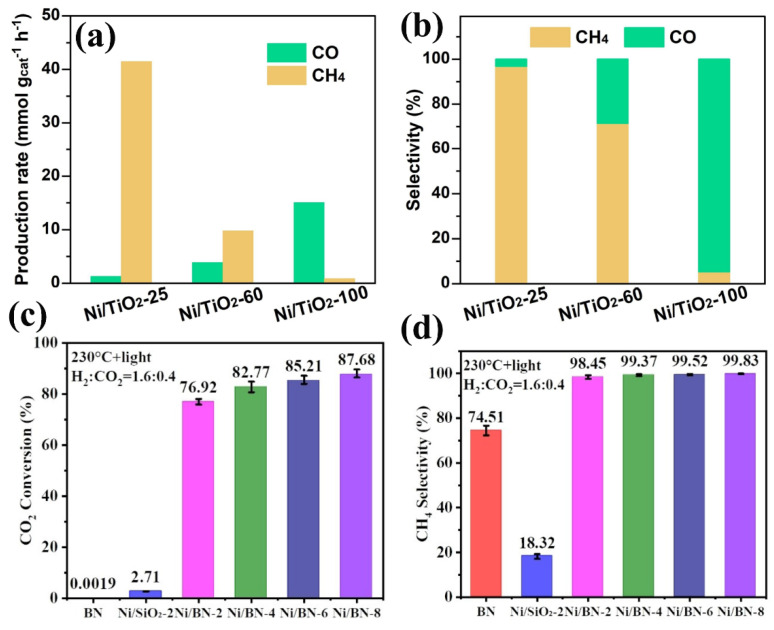
(**a**) The production rate and (**b**) selectivity images of the samples. (**c**) The CO_2_ conversion and (**d**) CH_4_ selectivity of the samples. Reprinted with permission from Ref. [78]. Copyright 2024, Wiley. Reprinted with permission from Ref. [79]. Copyright 2023, Wiley.

In addition, metal support interactions (MSIs) play an important role in regulating the photothermal CO_2_ methanation of Ni-based catalysts. For example, Li et al. used titanium dioxide supports of different sizes to effectively regulate the SMI between Ni and TiO_2_ [78]. It is found that the smaller size of TiO_2_ exhibits a stronger SMI. The main reason is that the smaller size of TiO_2_ has more sufficient oxygen vacancies on the surface, so the Ni atoms can be further effectively anchored, increasing the compatibility between the two. With the enhancement of SMI, photo-generated electrons by TiO_2_ excitation can easily migrate to the surface of Ni particles. The high electron density of Ni can promote the dissociation capacity of H_2_ molecules and the adsorption capacity of *CO intermediates, so that CO can undergo a deep hydrogenation reaction, resulting in the high selectivity of CH_4_. On the contrary, the SMI of large-sized TiO_2_ particles is weak, the adsorption capacity of the catalysts for *CO intermediates is insufficient, and it is easy for them to desorb and directly generate CO, resulting in the reduction in CH_4_ selectivity. As shown in Figure 4a,b, it can be clearly found that Ni/TiO_2_-25 (support size is about 25 nm) shows a higher CH_4_ selectivity than Ni/TiO_2_-100 (support size is about 100 nm). The reaction path is that TiO_2_ photoelectrons migrate to Ni particles to form Ni^−^ under ultraviolet–visible light irradiation; the left holes in TiO_2_ can assist H_2_ molecules to transform into H^+^ ions. Then, H^+^ combines with Ni^−^ to form Ni-H active species, which react with *CO_2_ to form many intermediates such as HCO*, H_2_CO*, H_3_CO* and so on. Finally, the C-O bonds of the above intermediates are broken to form CH_4_.

Moreover, the CO_2_ adsorption and activation of Ni-based catalysts are also important factors limiting the rate and selectivity of CO_2_ methanation. When the CO_2_ adsorption capacity of the catalyst is poor, CO_2_ will not be fully adsorbed on the active site, and the activation capacity of CO_2_ will be low, which will lead to an insufficient reaction concentration and a high CO_2_ conversion energy barrier, and may even cause changes in the type of intermediates and reaction path, thus limiting the reaction rate and selectivity. The developed “Frustrated Lewis Pairs” (FLPs) chemistry in recent years provides a useful approach and method for enhancing the design of CO_2_ chemisorption and activation capacities of catalysts. FLPs are generally composed of a pair of Lewis acid sites and Lewis base sites within or between molecules, but due to the obstruction of spatial coordination, these two active sites cannot form traditional Lewis acid–base admixtures, so they show some special chemical properties and catalytic activities. This theory gives us a new revelation: using this FLP chemical method constructs an active interface on the surface of the support, so as to significantly improve the CO_2_ adsorption and activation ability of the catalyst, which is a new strategy different from previous design ideas (the many existing methods are physical actions such as concepts of regulating the specific surface area and pore size, bonding activated carbon with a high CO_2_ adsorption capacity, adjusting the crystal plane, etc.). This method is promising to further improve the catalytic activity of CO_2_ methanation of nickel-based catalysts. Based on the above ideas, Jiang et al. constructed HOB···B FLPs on the surface of BN [79], and found that Ni/BN showed 87.68% CO_2_ methanation conversion, the reaction rate reached 2.03 mol g_Ni_^−1^ h^−1^, and the selectivity of CH_4_ was almost 100% (Figure 4c,d). The results show that FLPs can cooperate with Ni to capture and activate CO_2_ and H_2_, so as to perfectly convert CO_2_ into CH_4_.

### 3.3. Photothermal CO_2_ Hydrogenation for Methanol Production

**Figure 5:**
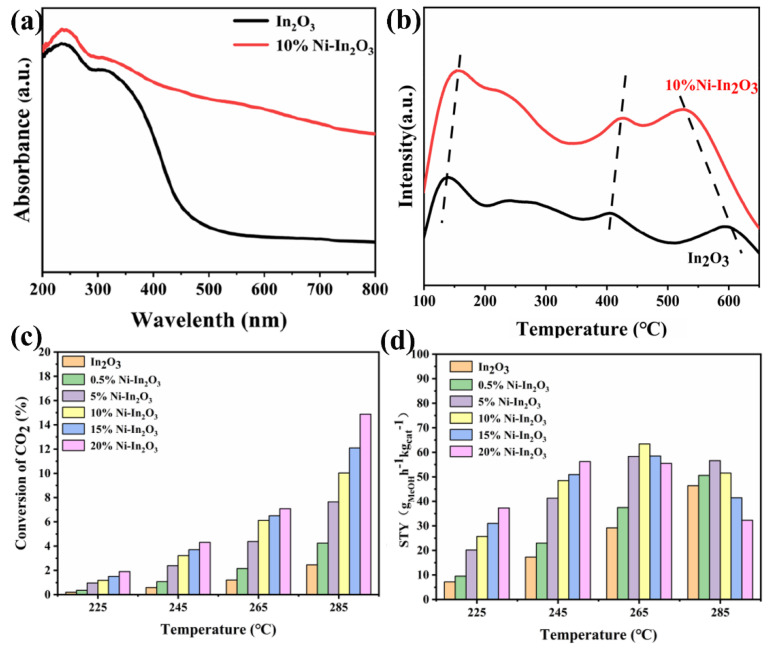
(**a**) The light absorption and (**b**) CO_2_ TPD images of In_2_O_3_ and 10%Ni-In_2_O_3_. (**c**) The CO_2_ conversion and (**d**) methanol yield of the samples. Reprinted with permission from Ref. [88]. Copyright 2024, American Chemical Society.

Methanol is one of the most important basic raw materials in the chemical industry, which is mainly used for the production of formaldehyde, dimethyl ether, acetic acid olefin (ethylene, propylene), aromatic (benzene, toluene, xylene), gasoline and other organic chemical products or fuels. It could partially alleviate the dependence on petroleum resources. Photothermal CO_2_ hydrogenation for methanol production can recycle carbon resources, thereby gradually eliminating the dependence on the decreasing fossil energy sources, which is of great significance to the sustainable development of human society. Ni-based catalysts are also a series of key materials for photothermal CO_2_ hydrogenation for methanol production. For example, Zhang et al. explored the application of Ni-In_2_O_3_ in photothermal CO_2_ hydrogenation for methanol production [88]. Firstly, Ni was doped into the crystal lattice of In_2_O_3_ using the co-precipitation method, and then, a highly dispersed distribution of Ni^0^ on the surface of In_2_O_3_ via reduction pretreatment was achieved. The introduction of Ni increased the oxygen vacancy concentration of In_2_O_3_ and expanded its light absorption capacity (Figure 5a). The increase in oxygen vacancy is conducive to the adsorption capacity of CO_2_ (Figure 5b), and the enhancement of its light absorption capacity can provide more energy and charge carriers for the reaction. Ni is in favor of H_2_ dissociation and can provide more H* active species for the reaction. Therefore, in the photothermal CO_2_ hydrogenation reaction, 10%Ni-In_2_O_3_ shows a higher CO_2_ conversion rate (Figure 5c) and a significantly increased methanol generation rate (Figure 5d) compared to pure In_2_O_3_. It can be seen from the above results that Ni can boost the methanol production rate of the catalyst with the reaction of photothermal CO_2_ hydrogenation for methanol production.

## 4. In Situ FT-IR Spectra Characterization

**Figure 6:**
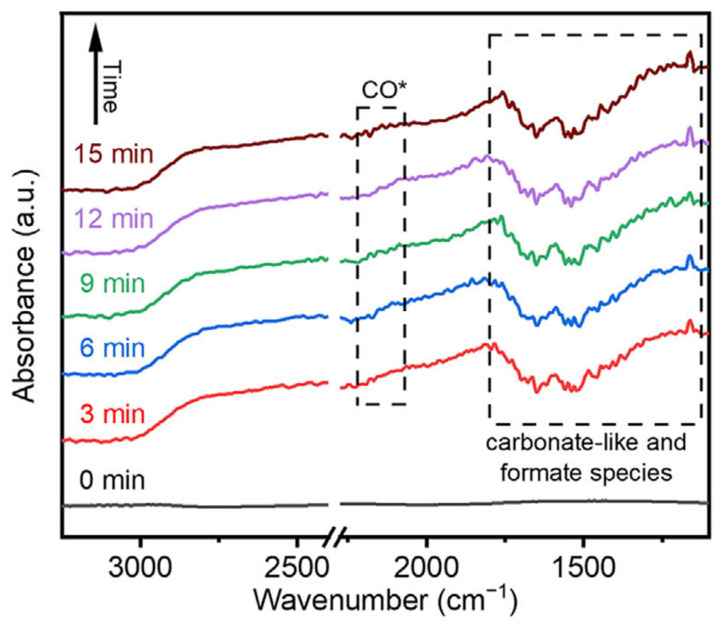
In situ FT-IR spectra of CO_2_ photothermal reduction of NiMo alloy in infrared reactor flowing under light irradiation. Reprinted with permission from Ref. [77]. Copyright 2024, Wiley.

In the photothermal CO_2_ hydrogenation reaction, a large number of Ni-based catalysts have been explored, but it is a great challenge to design them with the desired activity and selectivity due to the lack of understanding of the internal reaction process and the detailed mechanism of CO_2_ hydrogenation. In order to further explore the mystery of photothermal CO_2_ hydrogenation of Ni-based catalysts, in situ Fourier Transform infrared (FT-IR) spectra were used to detect the transient kinetic process of this reaction. Recently, Zhang et al. monitored the reaction process of NiMo alloys by using in situ FT-IR spectra while performing their photothermal CO_2_ hydrogenation test [77]. As shown in Figure 6, CO_2_ molecules adsorbed on the surface of NiMo alloys can be found to exist in the form of bicarbonate (HCO_3_^−^) species and monodentate carbonate (m-CO_3_^2−^). Their corresponding peaks are located at 1540 and 1650, and 1519, 1470 and 1704 cm^−1^. In addition, the peaks at 2110 cm^−1^ could be attributed to the *CO species, and the signals of *CH_x_ have not been detected throughout the supervision process. The in situ FT-IR spectra indicate that the photothermal CO_2_ hydrogenation process of NiMo alloys takes the *CO path, and the experimental results confirm the high CO selectivity mechanism. It can be seen from this study that in situ FT-IR spectra technology plays an important role in the in-depth study of the photothermal CO_2_ hydrogenation process of Ni-based catalysts, and it has become a necessary characterization method in this research field.

## 5. Future Perspectives and Summary

With the continuous progress of social productivity and science and technology, the energy crisis and environmental pollution has aroused great attention from all countries and governments, and they have put forward many coping strategies. Photothermal CO_2_ hydrogenation technology is considered a very good and promising solution to solve the above-mentioned environmental challenges. The key of the above application is suitable catalysts. Among all kinds of catalysts, Ni-based catalysts have become a series of hot-topic materials due to their advantages of low price, high catalytic activity, good stability and sufficient reserves. Their explorations in the field of photothermal CO_2_ hydrogenation have gradually deepened in recent years. Much research work has been conducted to further improve their photothermal catalytic efficiency and product selectivity for meeting practical application requirements. In this review, we summarized the relatively comprehensive photothermal CO_2_ hydrogenation applications of Ni-based catalysts, including photothermal CO_2_ methanation, the photothermal reverse water–gas reaction (RWGS), and photothermal CO_2_ hydrogenation for methanol production. Based on the above applications, this review presents the current research progress and the common reaction path. Many strategies have further enhanced the activities and product selectivity of Ni-based catalysts, but some current problems and challenges need be further studied or optimized, thereby directing future research.

(1) Currently, the main products of photothermal CO_2_ hydrogenation of Ni-based catalysts are CO, CH_4_ and CH_3_OH. With the deepening of research, it could be found that C_2+_ hydrocarbons are more valuable, but the current research progress and technical capabilities are not able to achieve this goal. In the explorations of some other classes of catalysts, related studies are gradually emerging. It can be observed that the main reaction process of C_2+_ hydrocarbon product production is the *CO-*CH_2_ pathway. *CO_2_ adsorbed on the catalyst surface forms *CO, which produces *CH_2_ by dissociation and hydrogenation of the C-O chemical bond. Then, *CH_2_ achieves C-C coupling under the action of a metal catalyst to obtain the generation of C_2+_ hydrocarbon products. Based on the above reaction path principle, Ni-based catalysts can be designed and explored to achieve the production of C_2+_ high-value-added hydrocarbons.
(2) Recently reported in the journal *Science*, when the catalyst in the CO_2_ hydrogenation reaction has 100% selectivity for CO and does not waste hydrogen to produce by-product methane, the RWGS reaction can better achieve the overall carbon negative benefit and simplify the downstream separation process. Therefore, the CO selectivity of Ni-based catalysts in the photothermal RWGS reaction needs to be improved. It is necessary to further modify the Ni-based catalyst or regulate the reaction system. In addition, some design ideas of organic catalysts can also be used for reference, which could do with some experimentation [89,90].
(3) The hydrogen spillover effect plays an important role in the photothermal CO_2_ hydrogenation reaction, but the current research mainly focuses on improving the catalytic activity by regulating the adsorption and activation capacities of CO_2_ and H_2_, and has neglected the hydrogen spillover effect. Therefore, in future studies, the mechanism of the hydrogen spillover effect should be explored in detail and applied for the improvement in the activity or selectivity of products.
(4) Due to the strong exothermic reaction and the eight-electron reduction process required for CO_2_ methanation, achieving its thermodynamic and kinetic reaction is a great challenge. The temperature is too low, the CO_2_ methanation reaction speed is slow, the high temperature will change the reaction balance and the CO_2_ conversion rate is low, which do not meet the needs of actual production. At present, few Ni-based catalysts can simultaneously meet the four high production needs of high rate, high conversion, high selectivity and high stability. Therefore, exploring and researching suitable low-temperature catalysts to reduce the energy barrier of CO_2_ conversion, accelerate the methane generation rate at a lower or more appropriate temperature, and ensure high CO_2_ conversion, high selectivity, high stability and low cost has become a key issue in this field.

## Data Availability

The data will be made available on request.
